# Clinical reasoning in canine meningoencephalitis of unknown origin and glioma: the role of signalment, history and neurological examination

**DOI:** 10.3389/fvets.2026.1920683

**Published:** 2026-10-09

**Authors:** Franziska Spohn, Samira Abani, Peter J. Dickinson, Karen M. Vernau, Christine M. Toedebusch, Kevin D. Woolard, Andreas Beineke, Holger A. Volk, Jasmin N. Nessler

**Affiliations:** 1 Department of Small Animal Medicine and Surgery, University of Veterinary Medicine Hannover, Foundation, Hannover, Germany; 2 Department of Surgical and Radiological Sciences, School of Veterinary Medicine, University of California, Davis, Davis, CA, United States; 3 Department of Pathology, Microbiology and Immunology, School of Veterinary Medicine, University of California, Davis, Davis, CA, United States; 4 Department of Pathology, University of Veterinary Medicine Hannover, Foundation, Hannover, Germany

**Keywords:** clinical reasoning, dogs, glioma, histopathology, meningoencephalitis of unknown origin (MUO)

## Abstract

**Introduction:**

Meningoencephalitis of unknown origin (MUO) is a group of presumed immune-mediated inflammatory diseases of the central nervous system (CNS) in dogs. Differentiating MUO from intracranial diseases such as glioma can be challenging based on magnetic resonance imaging (MRI) and cerebrospinal fluid (CSF) analysis. Because glioma represents an important differential diagnosis in dogs clinically suspected to have MUO, distinguishing these diseases is relevant for diagnostic reasoning, therapeutic planning, and owner communication. This study assessed whether readily available clinical variables contribute to clinical reasoning in dogs with histopathologically confirmed MUO or glioma.

**Methods:**

This retrospective multicenter study included 138 dogs with histopathologically confirmed MUO or glioma. Medical history, signalment, and neurological examination findings were compared between groups. Binary logistic regression was used to identify clinical variables associated with diagnosis.

**Results:**

Seventy dogs were diagnosed with MUO and 68 dogs with glioma. Dogs with MUO had a lower median body weight (10.50 kg vs. 23.90 kg; *p* = 0.0007) and age (5.00 years vs. 7.04 years; *p* = 0.0007) than dogs with glioma. In the final multivariable model, disease onset was associated with the distinction between MUO and glioma (*p* = 0.0282), while lower body weight and head tilt were associated with MUO. Increasing body weight was associated with lower odds of MUO (OR: 0.910; 95% CI: 0.861 – 0.961; *p* = 0.0007). Dogs without head tilt were less likely to be diagnosed with MUO than dogs with head tilt (OR: 0.249; 95% CI: 0.062 – 0.998; *p* = 0.0497). An exploratory, cohort specific body weight cut-off of ≤ 7.70 kg yielded 40.6% sensitivity and 95.6% specificity for MUO.

**Discussion:**

Readily available clinical variables may provide supportive information when MUO and glioma are differential diagnoses, particularly when MRI and CSF findings are equivocal. However, overlap between groups and limited discriminatory performance of individual variables indicate that these findings should not be used as standalone criteria. Instead, signalment, history, and neurological examination findings should be interpreted as part of an integrated assessment together with MRI, CSF analysis, and infectious testing.

## 1 Introduction

Meningoencephalitis of unknown origin (MUO) is a group of presumed immune-mediated inflammatory diseases of the central nervous system (CNS) in dogs (1, 2). Glioma represents an important differential diagnosis because both diseases may present as intra-axial brain lesions with overlapping magnetic resonance imaging (MRI) findings and in some cases, similar cerebrospinal fluid (CSF) abnormalities, making differentiation challenging without histopathological confirmation (3, 4). Accurate distinction between MUO and glioma is clinically relevant because therapeutic approaches, prognosis, and owner communication differ substantially between these diseases (5, 6).

Several studies have attempted to differentiate MUO from glioma based on MRI characteristics or CSF biomarkers (3, 7). MRI-based models have shown promising discriminatory performance, but misclassification may still occur, and diagnostic certainty remains limited in individual cases without histopathological confirmation (3). Although concentrations of various CSF biomarkers may differ between dogs with MUO and those with brain tumors, overlap between biomarker concentrations may limit their usefulness for differentiating individual dogs (7). Therefore, clinical reasoning in dogs with suspected MUO or glioma usually requires integration of all available information rather than reliance on a single diagnostic feature (2, 8).

Signalment, medical history and neurological examination findings are readily available at the beginning of the diagnostic process and are routinely incorporated into clinical reasoning. Clinical features of MUO and glioma have been described separately in previous studies (1, 2, 8, 9). However, direct comparisons of these readily available clinical variables between dogs with histopathologically confirmed MUO and glioma are limited. A direct comparison may help identify clinical variables that differ between these diseases and clarify which variables are independently associated with either diagnosis.

Therefore, the aim of the study was to compare signalment, history, and neurological examination findings between dogs with histopathologically confirmed MUO or glioma and to identify clinical variables independently associated with either diagnosis.

## 2 Material and methods

This retrospective multicenter study included dogs presented to the neurology services of the Small Animal Hospital of the University of Veterinary Medicine Hannover (TiHo) and the Small Animal Hospital of the UC Davis Veterinary Medical Teaching Hospital (VMTH) between 2006 and 2025. Cases were retrospectively identified from the clinical databases of both institutions. Dogs with a histopathologically confirmed diagnosis of cranial MUO or glioma were considered for inclusion. Dogs were included if the medical records contained information on signalment, medical history, and an initial neurological examination required to extract the predefined study variables. The neurological examination was performed by a Resident or Diplomate of the European College of Veterinary Neurology or American College of Veterinary Internal Medicine (Neurology).

Glioma diagnoses and grading were extracted from the original histopathology reports. Where available, gliomas were reported as oligodendroglioma, astrocytoma, or unclassified glioma and as low or high grade according to the terminology provided in the histopathology reports.

The following data were collected from the medical records: Breed, age at onset of the first clinical signs, sex, neuter status, body weight at the first referral, date of onset of clinical signs, date of referral, interval from onset to hospital presentation (days), survival time from onset (days), neurological examination findings, and epileptic seizure history. Due to the retrospective study design, not all variables were documented for every dog. Undocumented clinical findings were treated as missing values. Pain was classified as present when reported by the owner or identified during the neurological examination. Pain was classified as absent when available medical records documented no evidence of pain. If pain status could not be determined from the available medical records, it was treated as missing. Binary clinical and neurological variables were classified as normal or abnormal, based on the findings documented in the medical records.

For statistical analysis, German Shepherd Dogs, Belgian Shepherd Dog and Malinois were grouped as Shepherd dogs and French Bulldogs and English Bulldogs were grouped as Bulldogs. Onset of clinical signs was categorized as peracute (< 24 h), acute (1–7 days), subacute (8–14 days), chronic (> 14 days). Course of disease was defined as progressive, waxing and waning, or constant (unchanged, non-progressive); clinical signs were defined as symmetrical or asymmetrical, apparently painful (yes, no) and neuroanatomical localization was defined as either cerebrum, brainstem, cerebellar, spinal cord or multifocal.

Statistical analysis was done using SAS Studio Enterprise, Version 3.8.2 (SAS Institute Inc., Cary, North Carolina, USA).

For all variables, descriptive statistics were calculated. Continuous data are presented as mean ± standard deviation (SD) for normally distributed variables, and as median with interquartile range (IQR) for non-normally distributed variables. Normal distribution was assessed using the Shapiro-Wilk test. Continuous variables were compared between dogs with MUO and glioma using the Mann–Whitney *U* test, whereas categorical variables were compared using Pearson chi-square test or Fisher exact test, as appropriate. Because numerous univariate comparisons were performed, p-values were adjusted for multiple testing using the Benjamini–Hochberg false discovery rate (FDR) procedure. Where category-specific *post-hoc* comparisons were performed for categorical variables with more than two categories, these *p*-values were adjusted separately within each variable using the Benjamini–Hochberg FDR procedure.

Variables showing a significant association with disease group in the univariable analyses were subsequently grouped according to their clinical domain (signalment, clinical history, cranial nerve examination, gait, epileptic seizure-related variables) and evaluated in separate multivariable binary logistic regression models. Variables that remained significantly associated with disease group in the specific models were subsequently included as candidate variables in the final multivariable binary logistic regression model to identify independent variables associated with histopathological diagnosis of MUO or glioma. For categorical variables with more than two levels, the overall significance of the variable in the multivariable models was assessed using Type 3 Wald chi-square test. Dogs with missing data for any variables included in a respective logistic regression model were excluded from that analysis (complete-case analysis).

Results of the logistic regression analyses are reported as odds ratio (OR) with corresponding 95% confidence intervals (CI). Overall model significance was assessed using the likelihood-ratio test. Model fit was assessed using the Akaike Information Criterion (AIC), the Hosmer-Lemeshow goodness-of-fit test and the discriminatory ability of the final model was evaluated using the concordance statistic (c-statistic).

Receiver operating characteristic (ROC) analysis was performed for body weight, as it was the only continuous variable that remained independently associated with diagnosis in the final multivariable logistic regression model. The area under the curve (AUC) with corresponding 95% confidence intervals (CIs) was calculated. The exploratory cohort-specific threshold was determined by maximizing the Youden index (sensitivity + specificity – 1). Sensitivity and specificity at the selected threshold were reported with corresponding 95% CIs.

A *p*-value < 0.05 was considered statistically significant for all analyses. Unless otherwise stated, *p*-values reported for univariate comparisons are adjusted *p*-values obtained using the Benjamini–Hochberg false discovery rate procedure.

## 3 Results

A total of 138 dogs were included in this study. 70/138 dogs had the histopathological diagnosis of MUO and 68/138 were diagnosed with glioma. Within the MUO group, the histopathological diagnosis was granulomatous meningoencephalitis (GME) in 35/70 cases, undefined sterile lymphohistiocytic meningoencephalitis in 19/70 cases, and necrotizing encephalitis (NE), including necrotizing leukoencephalitis (NLE) and necrotizing meningoencephalitis (NME) in 16/70 cases. Among the 68 dogs with glioma, 46/68 were diagnosed with oligodendroglioma, including 22/46 high grade, 15/46 low grade and 9/46 unclassified cases. Astrocytoma was diagnosed in 10/68 dogs, including 1/10 high grade, 3/10 low grade and 6/10 cases unclassified. The remaining 12/68 dogs were classified as other glioma, comprising 7 glioblastoma multiforme, three unclassified glioma, one low grade glioma and one oligoastrocytoma based on the original histopathology reports.

### 3.1 Signalment

In the MUO group, 38/70 dogs were male (26/38 neutered) and 32/70 dogs were female (22/32 neutered). In the glioma group 42/68 dogs were male (35/42 neutered) and 26/68 dogs were female (21/26 neutered). At the onset of clinical signs, dogs with MUO were younger with a median age of 5.00 years (IQR: 4.02, range: 0.24–14.93 years) than dogs with glioma with a median age of 7.04 years (IQR: 3.62, range: 1.40–13.90 years; *p* = 0.0007; Table 1; Figure 1).

**Table 1:** Univariate comparison of significant differences in the signalment and history between meningoencephalitis of unknown origin (MUO) and glioma.

Variable	MUO	Glioma	*p* value
Age at onset (years), median (IQR; min–max), *n*	5.00 (4.02; 0.24–14.93), *n* = 68	7.04 (3.62; 1.40–13.90), *n* = 67	**0.0007**
Weight (kg), median (IQR; min–max), *n*	10.50 (19.10; 1.80–42.50), *n* = 69	23.90 (19.50; 3.40–63.00), *n* = 68	**0.0007**
Clinical signs until presentation (days), median (IQR; min–max), *n*	5.50 (12.00; 0.00–283.00), *n* = 68	20.00 (50.50; 0.00–426.00), *n* = 68	**0.0010**

IQR, interquartile range, calculated as Q3–Q1; n, number of dogs; significant level of *p* < 0.05. Bold *p*-values indicate significant overall differences between groups.

**Figure 1:**
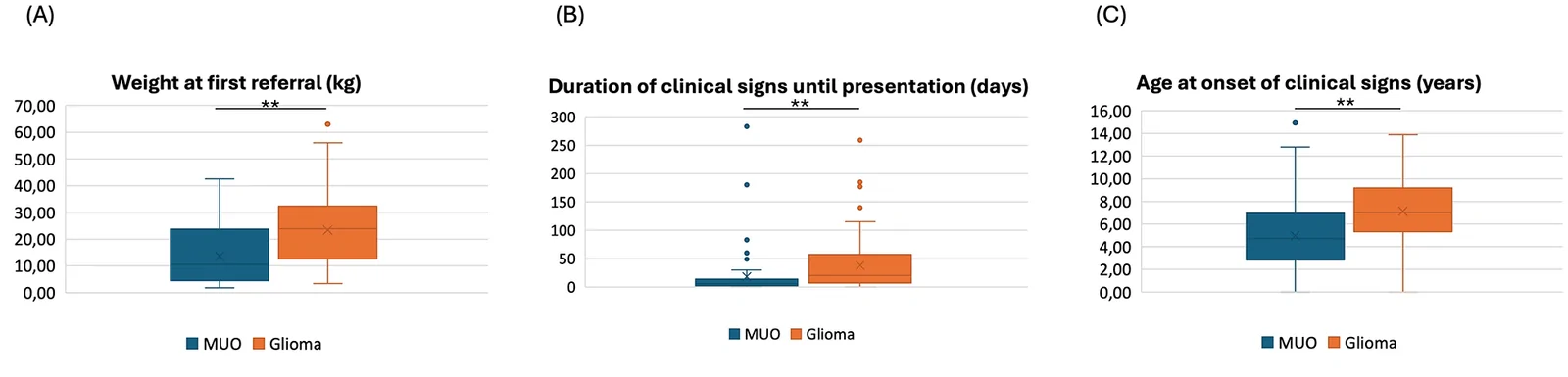
Box plots show the distribution of weight (kg) at first referral **(A)**, duration of clinical signs until presentation at the neurologist (days) **(B)**, and age at onset of clinical signs **(C)** in dogs with meningoencephalitis of unknown origin (MUO) compared with dogs with glioma. Asterisks indicate statistically significant differences between the groups (** *p* < 0.01). Boxes represent the interquartile range (25^th^ to 75^th^ percentiles) with a line indicating the median. An “X” within each box indicates the arithmetic mean; Whiskers show the most extreme values within 1.5x interquartile range, and observations beyond this range (outliers) are shown as individual points.

A total of 33 breeds were included. The most common breeds among dogs with MUO were: Mixed breed (*n* = 25), Yorkshire Terriers (*n* = 5), Australian Shepherd Dogs (*n* = 5), Chihuahuas (*n* = 5), Shepherd Dogs (*n* = 5), Bulldogs (*n* = 4), Pugs (n = 4), Maltese (*n* = 3), and West Highland White Terriers (*n* = 2). The most common breeds among dogs with glioma were: Bulldogs (*n* = 19), Boxers (*n* = 14), mixed breeds (*n* = 8), Boston Terriers (*n* = 5), Pit Bull Terriers (*n* = 4), Bullmastiffs (*n* = 2), Golden Retrievers (*n* = 2) and Labrador Retrievers (*n* = 2). Breed distribution differed between dogs with MUO and dogs with glioma (*p* = 0.0007). Descriptively, Bulldogs and Boxers were more common in the glioma group, whereas mixed breed dogs were more common in the MUO group. The median body weight was 10.50 kg (IQR: 19.10, range: 1.80–42.50 kg) in dogs with MUO, which was lower compared to dogs with glioma, whose median body weight was 23.90 kg (IQR: 19.50, range: 3.40 – 63.00 kg; *p* = 0.0007; Figure 1).

### 3.2 History

The onset of clinical signs was different between dogs with MUO and those with glioma (*p* = 0.0007). Dogs with MUO more often had an acute onset of clinical signs (1–7 days; *n* = 32/69; 46.38%), than dogs with glioma (*n* = 4/68; 5.88%; *p* = 0.0002). In contrast, dogs with glioma more frequently had a chronic history of clinical signs before presentation (> 14 days; *n* = 46/68; 67.65%) than dogs with MUO (*n* = 22/69; 31.88%; *p* = 0.0002; Figure 2). The median duration from onset to presentation was shorter for MUO (5.50 days; IQR: 12.00, range: 0.00–283.00 days) compared to glioma cases (20.00 days; IQR: 50.50, range: 0.00 – 426.00 days; *p* = 0.0010; Figure 1). The course of clinical signs was most commonly progressive in both groups. A progressive course was observed in 85.71% of dogs with MUO and 61.76% of dogs with glioma. Dogs with glioma more frequently showed a constant clinical course (*n* = 22/68, 32.35%) compared with dogs with MUO (n = 8/70, 11.43%; *p* = 0.0044; Figure 2).

**Figure 2:**
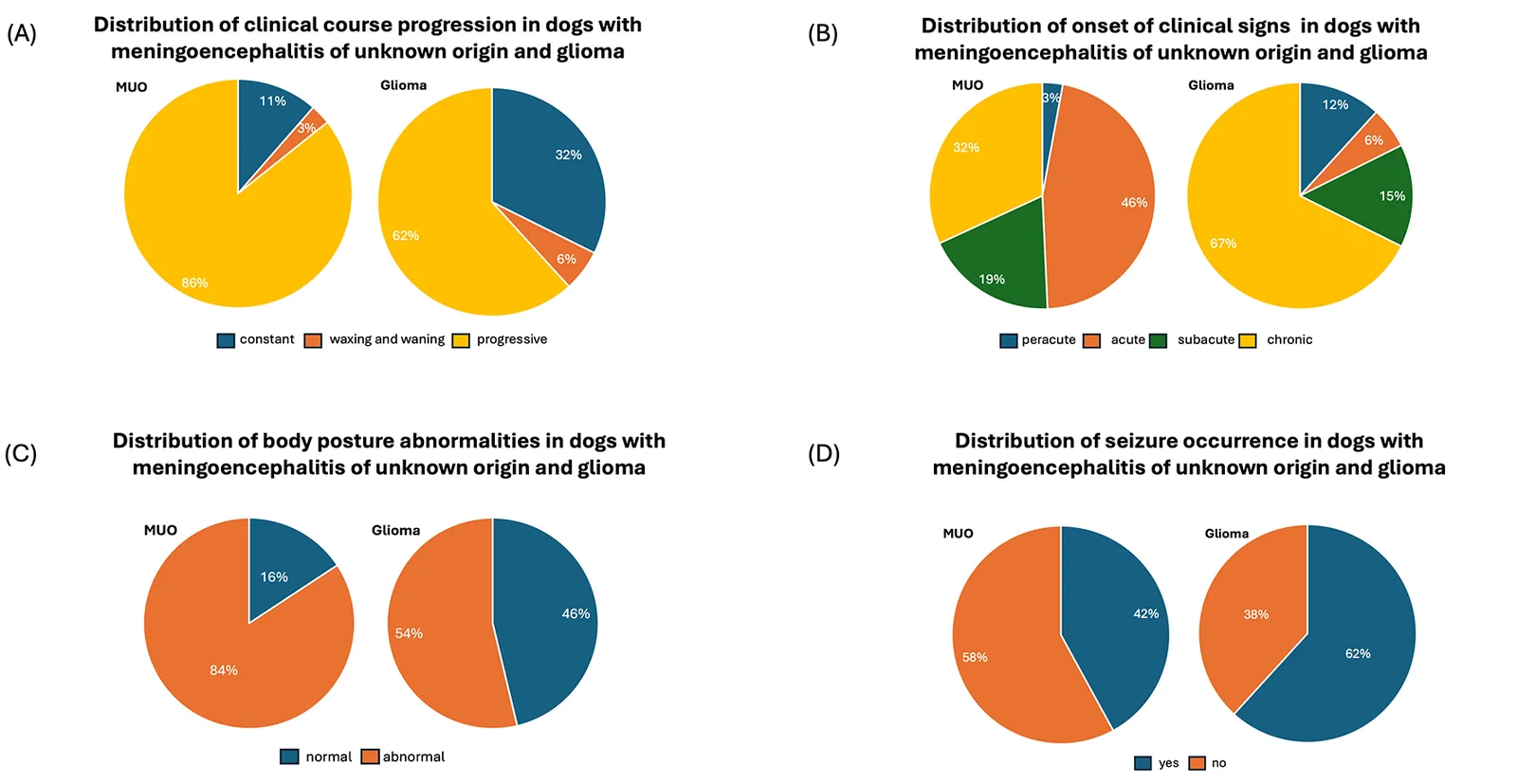
Pie charts showing the percentage distribution of progression of clinical signs **(A)**, onset of clinical signs **(B)**, presence of body posture abnormalities **(C)** and the occurrence of epileptic seizure **(D)** in dogs with meningoencephalitis of unknown origin (MUO) compared with dogs with glioma. Percentages are based on the number of dogs with available data for the respective variable. **(A)** Progression of clinical signs differed significantly between groups (*p* = 0.0122), with dogs with MUO more frequently showing a progressive course (*p* = 0.0042), whereas dogs with glioma more frequently showed a constant course (p = 0.0044). **(B)** The onset of clinical signs also varied significantly (*p* = 0.0007). MUO cases more commonly had an acute onset (*p* = 0.0002), whereas glioma cases more commonly had a chronic onset (*p* = 0.0002). **(C)** Abnormal body posture was observed more frequently in dogs with MUO, whereas dogs with glioma more frequently showed a normal body posture (*p* = 0.0022). **(D)** Epileptic seizures occurred more frequently in dogs with glioma than in dogs with MUO (*p* = 0.0402).

### 3.3 Neurological examination

Both groups most frequently showed an asymmetric presentation of clinical signs (MUO: 80.00%; glioma: 61.76%) and were most frequently reported as non-painful (MUO: 77.27%; glioma: 77.61%).

Neuroanatomical localization differed between groups (*p* = 0.0012). Cerebral localization was more common in dogs with glioma (*n* = 48/68, 70.59%) compared to dogs with MUO (*n* = 27/70, 38.57%; *p* = 0.0010) but was also the most frequently affected region in MUO cases. Multifocal intracranial involvement was more common in dogs with MUO (*n* = 23/70, 32.86%) than in dogs with glioma (n = 7/68, 10.29%, *p* = 0.0033; Table 2).

**Table 2:** Univariate comparison of clinical reasoning variables showing significant or frequent differences between meningoencephalitis of unknown origin (MUO) and glioma.

Variable	MUO (n/n)	Glioma (n/n)	p value
Onset			0.0007
Acute^*^	32/69 (46.38%)	4/68 (5.88%)	
Chronic^*^	22/69 (31.88%)	46/68 (67.65%)	
Progression			0.0122
Constant clinical course^*^	8/70 (11.43%)	22/68 (32.35%)	
Progressive^*^	60/70 (85.71%)	42/68 (61.76%)	
Symmetry			0.0377
Symmetric	14/70 (20.00%)	26/68 (38.24%)	
Asymmetric	56/70 (80.00%)	42/68 (61.76%)	
Pain			1.0000
Yes	15/66 (22.73%)	15/67 (22.39%)	
No	51/66 (77.27%)	52/67 (77.61%)	
Neuroanatomical localization			0.0012
Brainstem	18/70 (25.71%)	9/68 (13.24%)	
Cerebellum	0/70 (0.00%)	1/68 (1.47%)	
Cerebrum^*^	27/70 (38.57%)	48/68 (70.59%)	
Multifocal^*^	23/70 (32.86%)	7/68 (10.29%)	
Myelopathy	2/70 (2.86%)	3/68 (4.41%)	

Bold *p* value indicates significant overall differences between groups. ^*^ Indicates categories that were significant in post-hoc comparison after Benjamini–Hochberg correction within the respective variable.

Significant level of *p* < 0.05.

Mentation status differed between groups (*p* = 0.0222). Normal mentation was more common in dogs with glioma (*n* = 33/68, 48.53%) than in dogs with MUO (*n* = 16/67, 23.88%; *p* = 0.0064).

Body posture differed between the two groups (*p* = 0.0022). Dogs with glioma more frequently had a normal body posture (*n* = 31/67, 46.27%) than dogs with MUO (*n* = 11/62, 17.74%; Figure 2). Head tilt was more common in dogs with MUO (*n* = 23/64, 35.94%) than in dogs with glioma (n = 9/68, 13.24%; *p* = 0.0077), and head turn was also more frequently documented in dogs with MUO (*p* = 0.0377).

Most dogs in both groups were ambulatory (MUO: n = 41/65, 63.08%; glioma: *n* = 57/68, 83.82%) and frequently showed ataxia (MUO: *n* = 31/61, 50.82%; glioma: *n* = 28/65, 43.08%), whereas paresis was less frequently observed (MUO: *n* = 21/70, 30.00%; glioma: *n* = 17/68, 25.00%).

Cranial nerve deficits differed between the groups (*p* = 0.0010). Dogs with glioma were more likely to show normal cranial nerve examination (*n* = 32/66, 48.48%) than dogs with MUO (*n* = 12/66, 18.18%).

Epileptic seizures were more common in dogs with glioma (*n* = 42/68, 61.76%) than dogs with MUO (*n* = 29/69, 42.03%; *p* = 0.0402; Table 3, Figure 2).

**Table 3:** Univariate comparison of neurological examination variables showing significant or frequent differences between meningoencephalitis of unknown origin (MUO) and glioma.

Variable	MUO (n/n)	Glioma (n/n)	*p* value
Mentation			0.0222
Normal^*^	16/67 (23.88%)	33/68 (48.53%)	
Obtunded^*^	47/67 (70.15%)	31/68 (45.59%)	
Behavior change			0.2757
Normal	15/58 (25.86%)	25/67 (37.31%)	
Abnormal	43/58 (74.14%)	42/67 (62.69%)	
Body posture			0.0022
Normal	11/62 (17.74%)	31/67 (46.27%)	
Abnormal	51/62 (82.26%)	36/67 (53.73%)	
Head turn (Right/Left)			0.0377
Yes	18/64 (28.13%)	8/68 (11.76%)	
No	46/64 (71.88%)	60/68 (88.24%)	
Head tilt (Right/Left)			0.0077
Yes	23/64 (35.94%)	9/68 (13.24%)	
No	41/64 (64.06%)	59/68 (86.76%)	
Ataxia (type unspecified)			0.4843
Yes	31/61 (50.82%)	28/65 (43.08%)	
No	30/61 (49.18%)	37/65 (56.92%)	
Ambulatory			0.0174
Yes	41/65 (63.08%)	57/68 (83.82%)	
No	24/65 (36.92%)	11/68 (16.18%)	
Paresis			0.5926
Yes	21/70 (30.00%)	17/68 (25.00%)	
No	49/70 (70.00%)	51/68 (75.00%)	
Cranial nerve deficits			0.0010
Yes	54/66 (81.82%)	34/66 (51.51%)	
No	12/66 (18.18%)	32/66 (48.48%)	
Epileptic seizure			0.0402
Yes	29/69 (42.03%)	42/68 (61.76%)	
No	40/69 (57.97%)	26/68 (38.24%)	

Bold *p* value indicates significant overall differences between groups. ^*^ Indicates categories that were significant in post-hoc comparison after Benjamini–Hochberg correction within the respective variable.

Significant level of *p* < 0.05. Not all recorded mentation categories are displayed in the table.

The complete results of the univariable analyses are presented in Supplementary Table S1.

Based on the exploratory analysis and variable selection described in the statistical analysis section, nine variables were included in the final multivariable binary logistic regression model. Model significance was confirmed by the likelihood-ratio test (χ^2^ = 69.02, *df* = 11, *p* < 0.00001), and model fit was acceptable based on the Hosmer-Lemeshow goodness-of-fit test (χ^2^ = 6.36, *df* = 8, *p* = 0.6068). The final model showed an apparent discriminatory performance (c-statistic of 0.902). As this estimate was derived from the development dataset, it represents the apparent model performance and has not been internally validated.

Result of the specific multivariable logistic regression models used for variable selection are provided in Supplementary Table S2. A total of 120 dogs (56 MUO and 64 glioma) were included in the final multivariable binary logistic regression model. Disease onset was associated with the distinction between MUO and glioma (*p* = 0.0282). However, none of the individual onset categories differed significantly from the reference category (subacute). Acute onset of clinical signs showed the strongest association with MUO (β = 1.6058; 95% CI: 0.949 – 26.151; OR: 4.982, *p* = 0.0577). Increasing body weight was associated with lower odds of MUO (β =-0.0948; 95% CI: 0.861 – 0.961; OR: 0.910, *p* = 0.0007). Dogs without head tilt were less likely to be diagnosed with MUO compared with dogs with head tilt (β =-1.3912; 95% CI: 0.062 – 0.998; OR: 0.249, *p* = 0.0497) (Table 4).

**Table 4:** Final multivariable binary logistic regression model identifying clinical variables associated with meningoencephalitis of unknown origin (MUO) compared with glioma.

Variable	Odds ratio	95% Confidence interval	Coefficient (β)	*p*- value
Onset				0.0282
Acute	4.982	0.949–26.151	1.6058	0.0577
Chronic	0.567	0.143–2.254	−0.5667	0.4207
Peracute	0.346	0.037–3.240	−1.0606	0.3526
Signalment				
Age onset (days)	1.000	0.999–1.000	−0.00045	0.0775
Weight (kg)	0.910	0.861–0.961	−0.0948	0.0007
Clinical history				
Clinical signs until presentation (days)	0.995	0.983–1.006	−0.00513	0.3825
Neurological examination				
Body posture (abnormal)	1.667	0.415–6.700	0.5108	0.4718
Head tilt (no)	0.249	0.062–0.998	−1.3912	0.0497
Cranial nerve deficits (no)	0.669	0.211–2.127	−0.4016	0.4961
Seizure at any point (no)	1.383	0.403–4.750	0.3240	0.6068
Ambulatory (no)	1.220	0.334–4.452	0.1988	0.7634

The model outcome was MUO. Odds ratio > 1 indicate higher odds of MUO, whereas odds ratios < 1 indicate lower odds of MUO compared with glioma. Significant level of *p* < 0.05.

Exploratory ROC analysis of body weight yielded an AUC of 0.741 (95% CI: 0.659 – 0.823). The exploratory, cohort-specific body weight threshold using the Youden index was ≤ 7.70 kg. At this threshold, sensitivity was 40.6% (95% CI: 28.9 – 53.1%) and specificity was 95.6% (95% CI 87.6 – 99.1%).

## 4 Discussion

This study identified several readily available clinical variables that may contribute to clinical reasoning in dogs with histopathologically confirmed MUO and glioma. In the final model, disease onset was associated with the distinction between MUO and glioma, while lower body weight and head tilt were associated with MUO. These findings suggest that signalment, history, and selected neurological examination findings can provide useful contextual information during diagnostic assessment, particularly when interpreted together with MRI, CSF analysis and other diagnostic findings.

Dogs with MUO had a lower median age than dogs with glioma in this study (5.00 years vs. 7.04 years), which is consistent with previous descriptions of MUO and glioma populations (2, 8–11). However, the overlap between the groups and the lack of independent contribution in the final model suggest that age alone has limited value for differentiating MUO from glioma in individual dogs.

Breed and body weight should be interpreted together, as they are biologically related. Boxers and Boston Terriers were only represented in the glioma group, and Bulldogs were also more common among dogs with glioma. These findings are consistent with previous studies reporting a predisposition of brachycephalic breeds, particularly Boxers, Bulldogs, and Boston Terriers to canine glioma, especially oligodendroglioma (8, 9, 12). In contrast, mixed breed dogs and smaller dogs were more frequently represented among dogs with MUO, which is consistent with previous descriptions of MUO populations (1, 2, 10). The higher body weight observed in dogs with glioma may partly reflect the breed distribution of the glioma group, as breeds such as Boxers, Bulldogs, and Bullmastiffs are generally heavier than many breeds commonly represented in MUO populations. Whether breeds predisposed to glioma are underrepresented among dogs with MUO cannot be determined from the present study and would require population-based data. Therefore, signalment, including breed and body weight, may provide useful contextual information during clinical reasoning, but these variables are not independent and should not be interpreted as standalone discriminatory criteria. In addition, the exploratory body weight threshold showed only low sensitivity but high specificity, indicating that body weight alone is insufficient for clinical decision making and should be interpreted together with other clinical findings. Furthermore, the observed association between body weight and diagnosis may be cohort dependent and partly reflect the breed composition of the present study population.

Dogs with MUO were presented to a neurologist earlier after the onset of neurological signs than dogs with glioma. In this study, MUO was more often associated with an acute onset of clinical signs (1–7 days), whereas glioma was more frequently associated with a chronic onset (> 14 days). These observations are consistent with previous studies, in which dogs with glioma were presented after a median of 14 days following the onset of signs (8). This delayed presentation in glioma cases may reflect the slower growth and slower progression of these tumors. In addition, dogs with glioma may be presented after a first observed epileptic seizure, which is often the earliest clinical sign of a glioma (8, 13, 14). It is conceivable that some owners may have perceived subtle or intermittent clinical signs before presentation, which only became apparent in retrospect following veterinary examination. However, epileptic seizures are not specific for glioma and were also observed in dogs with MUO in the present study. Epileptic seizures have also been reported in up to 33% of the dogs with MUO in previous studies (15, 16). Therefore, epileptic seizure occurrence should be interpreted together with overall clinical course, neuroanatomical localization, MRI findings, and CSF results rather than as an isolated discriminator between MUO and glioma.

In contrast, dogs with MUO, particularly those with multifocal inflammatory involvement, often showed an acute onset of clinical signs (1, 10). Multifocal neurological involvement has been reported in approximately 40–50% of dogs with MUO, whereas focal signs have been described less frequently in approximately 18% of cases (1, 2, 17). This distribution is consistent with the present study in which multifocal localization was more frequently observed in dogs with MUO than in dogs with glioma. These findings may explain why some dogs with MUO presented with a combination of neurological deficits including ataxia, vestibular dysfunction, visual deficits, or epileptic seizures (18).

The distribution of neuroanatomical localization provides a plausible explanation for these clinical patterns observed in this study. MUO cases were commonly localized as multifocal (32.86%) or cerebral (38.57%), whereas glioma cases were mainly localized within the cerebrum (70.59%). The predominance of cerebral localization in glioma is consistent with previous reports describing the cerebral hemispheres as the most common site for canine gliomas (8, 12, 19). Conversely, the higher frequency of multifocal involvement in dogs with MUO is consistent with the potential for inflammatory CNS disease to affect the multiple neuroanatomical regions (1, 2). From a clinical perspective, multifocal localization together with a progressive clinical course may contribute to the suspicion for MUO, whereas more constant clinical course combined with focal cerebral signs may contribute to the suspicion of glioma. However, these findings should be interpreted as part of the broader clinical pattern rather than as independent predictors, because neuroanatomical localization, disease course, and individual neurological signs are clinically related and were not all retained in the final model.

Among the neurological examination findings, head tilt was the most relevant variable retained in the final model and was associated with MUO. This finding is clinically plausible, as head tilt is typically associated with vestibular system dysfunction, which may involve either peripheral or central vestibular structures. In dogs with MUO, inflammatory lesions may affect the brainstem, cerebellum or multiple neuroanatomical regions (1, 2), which may explain the higher prevalence of vestibular signs in this group compared with gliomas.

In summary, the variables identified in this study may support clinical reasoning when MUO and glioma are considered as differential diagnoses. The pattern of disease onset, lower body weight, and head tilt may provide useful information when considering MUO as a differential diagnosis, particularly when they occur together and are interpreted alongside MRI, CSF analysis and infectious disease testing. However, because substantial overlap exists between MUO and glioma, these variables should be interpreted as supportive clinical information rather than standalone criteria for diagnosis.

Several potential sources of bias should be considered when interpreting the results of this study. First, the retrospective multicenter design may have introduced variability in the documentation of clinical signs, onset, progression and apparent pain. There were no uniform criteria for assessing parameters such as pain or the onset of clinical signs, which may have led to inconsistencies and subjective recording of disease course or description of clinical signs.

Second, inclusion required histopathological confirmation, which was most often obtained postmortem. This may have introduced selection bias toward dogs with more severe clinical signs, more rapid disease progression, or poor outcome. Dogs with MUO that responded well to treatment and survived long term were less likely to be included and may therefore be underrepresented in this study. In addition, dogs with MUO and multifocal signs may tend to have a worse prognosis (20).

Third, potential statistical bias should be considered. Several variables included in the analysis were clinically related, and complete independence between variables cannot be assumed. Examples include breed and body weight, as well as neuroanatomical localization and neurological signs such as head tilt, cranial nerve deficits, and epileptic seizure history. Although the variable selection process followed the predefined statistical approach described in the methods, the modeling strategy remained exploratory. Therefore, overfitting, false-positive findings, and residual dependence between variables cannot be excluded, and the multivariable regression results should be interpreted cautiously.

Finally, some histopathological subgroups of MUO and glioma, such as NE or astrocytoma, were represented only by a small number of cases. Because MUO and glioma were analyzed as broad disease categories, potential subtype-specific differences may not have been detected, and the results may be weighted toward the more common subtypes, such as GME or oligodendroglioma. This may be relevant because MUO subtypes can differ in their neuroanatomical distribution; for example, a predominance of GME could increase the frequency of multifocal, brainstem or cerebellar signs, whereas a higher proportion of NE might have resulted in a more cerebral clinical pattern (1, 2, 18). Similarly, the predominance of oligodendroglioma may limit extrapolation of the glioma findings to less frequent glioma subtypes such as astrocytoma.

Furthermore, the body weight threshold identified by ROC analysis should be interpreted as exploratory and cohort specific because it was derived and evaluated in the same study population. External validation in an independent cohort is required before it can be considered for broader clinical application.

## 5 Conclusion

Readily available clinical variables may support clinical reasoning in dogs with histopathologically confirmed MUO or glioma. In the final model, disease onset was associated with the distinction between MUO and glioma, while lower body weight and the presence of head tilt were independently associated with MUO. These readily assessable clinical features may provide supportive contextual information during clinical reasoning but should not be used as standalone diagnostic criteria.

However, the observed overlap between groups indicates that these variables alone are not sufficient for diagnosis or treatment decisions in an individual dog. The results should therefore be interpreted within the context of an integrated diagnostic assessment and as a retrospective comparison of two predefined disease groups, rather than as a substitute for evaluating the full range of clinically relevant differential diagnoses.

## Data Availability

The data analyzed in this study is subject to the following licenses/restrictions: the original datasets generated and analyzed during the current study are not included in the article and are not publicly available because they contain information derived from clinical patient records; Requests to access these datasets should be directed to F. Spohn, franziska.spohn@tiho-hannover.de.
