# Supplementary material for: Clinical reasoning in canine meningoencephalitis of unknown origin and glioma: the role of signalment, history and neurological examination
Source: Front Vet Sci. 2026 Oct 9;13:1920683. doi: 10.3389/fvets.2026.1920683 (PMC13692783; doi:10.3389/fvets.2026.1920683)
Supplement: Supplementary file 2 [file Table_2.docx]

Supplementary Table S1: **Results of univariable comparisons between dogs with histopathologically confirmed meningoencephalitis of unknown origin (MUO) and glioma.**

|  |  | **MUO** | **Glioma** | **p value** |
| --- | --- | --- | --- | --- |
| **Number of dogs, n** |  | n = 70 | n = 68 |  |
| **Gender, n** |  | M: n = 38, F: n = 32 | M: n = 42; F: n = 26 | p = 0.4843 |
| **Neuter status, n** |  |  |  | p = 0.1093 |
|  | Male | No: n = 12; Yes: n = 26 | No: n = 7; Yes: n = 35 |  |
|  | Female | No: n = 10; Yes: n = 22 | No: n = 5; Yes: n = 21 |  |
| **age of onset (years), median (min - max), n** |  | 5.00 (0.24 - 14.93), n = 68 | 7.04 (1.40 - 13.90), n= 67 | p = 0.0007 |
| **Breed, n** |  |  |  | p = 0.0007 |
|  | Most common breeds | Xbreed*: n = 25;  Yorkshire Terrier: n = 5;  Australian Shepherd: n = 5;  Chihuahua: n = 5;  Shepherd Dog: n = 5;  Bulldog: n = 4;  Pug: n = 4;  Maltese: n = 3;  West Highland White Terrier: n = 2; | Bulldog: n = 19;  Boxer*: n = 14;  Xbreed: n = 8;  Boston Terrier: n = 5;  Pit Bull Terrier: n = 4;  Golden Retriever: n = 2;  Labrador Retriever: n = 2;  Bullmastiff: n = 2; |  |
|  | Other breed n = 1 each | Airedale Terrier, Australian Cattle Dog, Bichon Frise, Border Collie, Cairn Terrier, Dachshund, Dalmatian, Labrador Retriever, Podenco Ibicenco, Pomeranian, Poodle, Shih tzu, | Australian Shepherd, Border Terrier, Chihuahua, Doberman Pinscher, Goldendoodle, Great Dane, Jack Russell Terrier, Miniature Poodle, Siberian Husky, Staffordshire Bull Terrier, Shepherd dog |  |
| **Weight (kg),**  **median (min - max), n** |  | 10.50 (1.80-42.50), n = 69 | 23.90 (3.40- 63.00), n = 68 | p = 0.0007 |
| **Clinical signs until presentation (days), median (min-max), n** |  | 5.50 (0.00- 283.00), n = 68 | 20.00 (0.00- 426.00), n = 68 | p = 0.0010 |
| **onset** |  |  |  | p = 0.0007 |
|  | peracute | n = 2/69 (2.90 %) | n = 8/68 (11.76 %) |  |
|  | acute* | n = 32/69 (46.38 %) | n = 4/68 (5.88 %) |  |
|  | subacute | n = 13/69 (18.84 %) | n = 10/68 (14.71 %) |  |
|  | chronic* | n = 22/69 (31.88 %) | n = 46/68 (67.65 %) |  |
| **progression** |  |  |  | p = 0.0122 |
|  | constant clinical course* | n = 8/70 (11.43 %) | n = 22/68 (32.35 %) |  |
|  | waxing and waning | n = 2/70 (2.86 %) | n = 4/68 (5.88 %) |  |
|  | progressive* | n = 60/70 (85.71 %) | n = 42/68 (61.76 %) |  |
| **symmetry** |  |  |  | p = 0.0377 |
|  | symmetric | n = 14/70 (20.00 %) | n = 26/68 (38.24 %) |  |
|  | asymmetric | n = 56/70 (80.00 %) | n = 42/68 (61.76 %) |  |
| **pain** |  |  |  | p = 1.0000 |
|  | Yes | n = 15/66 (22.73 %) | n = 15/67 (22.39 %) |  |
|  | No | n = 51/66 (77.27 %) | n = 52/67 (77.61 %) |  |
| **Neuroanatomical localization** |  |  |  | p = 0.0012 |
|  | Brainstem | n = 18/70 (25.71 %) | n = 9/68 (13.24 %) |  |
|  | Cerebellum | n = 0/70 (0.00 %) | n = 1/68 (1.47 %) |  |
|  | Cerebrum* | n = 27/70 (38.57 %) | n = 48/68 (70.59 %) |  |
|  | Multifocal* | n = 23/70 (32.86 %) | n = 7/68 (10.29 %) |  |
|  | Myelopathy | n = 2/70 (2.86 %) | n = 3/68 (4.41 %) |  |
| **Mentation** |  |  |  | p = 0.0222 |
|  | normal* | n = 16/67 (23.88 %) | n = 33/68 (48.53 %) |  |
|  | obtunded* | n = 47/67 (70.15 %) | n = 31/68 (45.59 %) |  |
|  | epileptic seizure | n = 0/67 (0.00 %) | n = 0/68 (0.00 %) |  |
|  | stoic | n = 1/67 (1.49 %) | n = 1/68 (1.47 %) |  |
|  | stuporous | n = 2/67 (2.99 %) | n = 3/68 (4.41 %) |  |
| **Behavior change** |  |  |  | p = 0.2757 |
|  | normal | n = 15/58 (25.86 %) | n = 25/67 (37.31 %) |  |
|  | abnormal | n = 43/58 (74.14 %) | n = 42/67 (62.69 %) |  |
| **Body posture** |  |  |  | p = 0.0022 |
|  | normal | n = 11/62 (17.74 %) | n = 31/67 (46.27 %) |  |
|  | abnormal | n = 51/62 (82.26 %) | n = 36/67 (53.73 %) |  |
| **Head turn (Right/Left)** |  |  |  | p = 0.0377 |
|  | No | n = 46/64 (71.88 %) | n = 60/68 (88.24 %) |  |
|  | Yes | n = 18/64 (28.13 %) | n = 8/68 (11.76 %) |  |
| **Head tilt (Right/Left)** |  |  |  | p = 0.0077 |
|  | No | n = 41/64 (64.06 %) | n = 59/68 (86.76 %) |  |
|  | Yes | n = 23/64 (35.94 %) | n = 9/68 (13.24 %) |  |
| **Tremor** |  |  |  | p = 1.0000 |
|  | Yes | n = 1/50 (2.00 %) | n = 1/67 (1.49 %) |  |
|  | No | n = 49/50 (98.00 %) | n = 66/67 (98.51 %) |  |
| **Dysmetria** |  |  |  | p = 0.1472 |
|  | Yes | n = 7/60 (11.67 %) | n = 2/65 (3.08 %) |  |
|  | No | n = 53/60 (88.33 %) | n = 63/65 (96.92 %) |  |
| **Vestibular ataxia** |  |  |  | p = 0.3008 |
|  | Yes | n = 4/61 (6.56 %) | n = 1/65 (1.54 %) |  |
|  | No | n = 57/61 (93.44 %) | n = 64/65 (98.46 %) |  |
| **Cerebellar ataxia** |  |  |  | p = 0.5850 |
|  | Yes | n = 1/61 (1.64 %) | n = 0/65 (0.00 %) |  |
|  | No | n = 60/61 (98.36 %) | n = 65/65 (100.00 %) |  |
| **Proprioceptive ataxia** |  |  |  | p = 1.0000 |
|  | Yes | n = 1/61 (1.64 %) | n = 1/65 (1.54 %) |  |
|  | No | n = 60/61 (98.36 %) | n = 64/65 (98.46 %) |  |
| **Ataxia (unknown)** |  |  |  | p = 0.4843 |
|  | Yes | n = 31/61 (50.82 %) | n = 28/65 (43.08 %) |  |
|  | No | n = 30/61 (49.18 %) | n = 37/65 (56.92 %) |  |
| **Ambulatory** |  |  |  | p = 0.0174 |
|  | Yes | n = 41/65 (63.08 %) | n = 57/68 (83.82 %) |  |
|  | No | n = 24/65 (36.92 %) | n = 11/68 (16.18 %) |  |
| **Paresis** |  |  |  | p = 0.5926 |
|  | Yes | n = 21/70 (30.00 %) | n = 17/68 (25.00 %) |  |
|  | No | n = 49/70 (70.00 %) | n = 51/68 (75.00 %) |  |
| **Plegia** |  |  |  | p = 0.3379 |
|  | Yes | n = 3/70 (4.29 %) | n = 0/68 (0.00 %) |  |
|  | No | n = 67/70 (95.71 %) | n = 68/68 (100.00 %) |  |
| **Postural reaction** |  |  |  | p = 0.3379 |
|  | absent | n = 39/55 (70.91 %) | n = 36/60 (60.00 %) |  |
|  | delayed | n = 9/55 (16.36 %) | n = 9/60 (15.00 %) |  |
|  | normal | n = 7/55 (12.73 %) | n = 15/60 (25.00 %) |  |
| **head nerve involvement** |  |  |  | p = 0.0010 |
|  | Yes | n = 54/66 (81.82 %) | n = 34/66 (51.51 %) |  |
|  | No | n = 12/66 (18.18 %) | n = 32/66 (48.48 %) |  |
| **Paroxysmal dyskinesia** |  |  |  | p = 1.0000 |
|  | Yes | n = 3/70 (4.29 %) | n = 2/68 (2.94 %) |  |
|  | No | n = 67/70 (95.71 %) | n = 66/68 (97.06 %) |  |
| **Epileptic Seizure** |  |  |  | p = 0.0402 |
|  | Yes | n = 29/69 (42.03 %) | n = 42/68 (61.76 %) |  |
|  | No | n = 40/69 (57.97 %) | n = 26/68 (38.24 %) |  |

**Continuous variables are presented as median (minimum – maximum) or mean ± SD, as appropriate.**

**Categorical variables are presented as number (%).**

**P-values for overall univariable comparison were adjusted for multiple testing using the Benjamini-Hochberg false discovery rate (FDR) procedure unless otherwise stated.**

***Indicates categories that were significant in post-hoc comparison after Benjamini-Hochberg correction** within the respective variable**.**

**n: number of dogs**
